# Supplementary material for: Patient and Family Financial Burden in Cancer: A Focus on Differences across Four Provinces, and Reduced Spending Including Decisions to Forego Care in Canada
Source: Curr Oncol. 2024 May 11;31(5):2713–26. doi: 10.3390/curroncol31050206 (PMC11119025; doi:10.3390/curroncol31050206)
Supplement: Supplementary file 1 [file curroncol-31-00206-s001.zip › curroncol-2760344-supplementary.pdf]

Supplementary Materials

Patient- Self-Administered Financial Effects (P-SAFE) Questionnaire

P-SAFE (General Cancer)

Version 7.2.4

May 31st, 2018

Introduction

This questionnaire will ask about the costs for you (as patient) related to a diagnosis of cancer.

In total, there are **4 parts** with a total of **31 questions**. **Please answer all questions**.

Part 1:

Questions 1 to 11 will ask about insurance plans that help pay for health services as well as additional costs you had for your care. Here we will ask about **extra costs**, for example,

- Costs when you were in the hospital
- or money needed to pay for medicines
- or in-home health services or other health professionals.

Please note costs for caregivers (for example, spouse , children or parents) are **not included** here.

Part 2:

Questions 12 to 14 will ask about time away from work for yourself [and caregiver(s)] because of *your* cancer diagnosis.

Part 3:

Questions 15 to 30 ask about some important information about you and your treatment.

Part 4:

Question 31 will ask you about any other costs **not included** in Parts 1 or 2.

Additional instruction for Question 1:

What we mean by the following?

"**Full coverage**"	means that someone else pays for **all** of that service for you.
"**Partial coverage**"	means that someone else pays for **some**, but not all of that service for you.
"**No coverage**"	means that **you pay for all** of that service yourself.
**"Not applicable or NA"**	means that you **don't use** this service.

Part 1: Questions 1 to 11 ask about coverage and cancer-related costs for your care.

Over the **PAST 4 weeks**, please share who **has or would have helped** you pay for **your cancer-related costs**. Please include private and/or government insurance (e.g. Trillium). For example, costs include extra costs for your care when you were in the hospital OR medicines or home health services or other health professionals at home.

If you do NOT have private or employer paid health insurance ☞ Go to Question 3

1. If you have Private/Employer-paid health insurance, please describe your coverage for each type of service: (For each service, check the box that best describes your level of coverage.)

TYPE OF SERVICE	✓*Don't Know*	✓*Not Covered*	✓*Some Coverage*	✓*Full Coverage*	**Employer/work PAID**[1]
Hospital supplemental/upgrade charges (e.g. Private room, telephone, TV, etc.)					*Y or N*
Prescription drugs (e.g. Antibiotics, pain medication, etc.)					*Y or N*
In home healthcare (e.g. nursing, physiotherapist, etc.)					*Y or N*
Homemaking services (e.g. cleaning, cooking, etc.)					*Y or N*
Alternate Therapy (e.g. Homeopathy, Chinese medicine, over the counter drugs)					*Y or N*
Disability insurance					*Y or N*
Long term care insurance[2]					*Y or N*
CRITICAL ILLNESS INSURANCE[3]					*Y or N*
Other (Specify) ___________________________________					*Y or N*

1. If your health insurance at work pays for any of the above listed services then choose YES for "premium employer PAID"
2. Long term care insurance is an insurance package that reimburses some of the costs for long term care or nursing home services.
3. Critical illness insurance is an insurance package that pays off a fixed amount of money (the cash payout) in case of a diagnosed illness such as cancer.

2. If you DO HAVE critical illness insurance what is the cash payout of your critical illness insurance (How much did you receive or are you going to receive from your insurer for your cancer)? ❑ **$ _____________** ❑ Don't know

☞ *If no Critical Illness Insurance Go to Question 3*

2b: Based on your experience how much critical illness insurance would you suggest a patient purchase:

❑ A higher cash payout than myself (specify preferred amount) $ _______________
❑ A lower cash payout than myself (specify preferred amount) $ _______________
❑ About the same amount of cash payout as myself

2c: How do you think you will be (or you are) using the critical illness insurance money (check ALL that apply)?

❑ use it to pay existing debt ❑ Use it to pay for care ❑ use it to cover basic living expenses
❑ Use it for vacation or other leisure purchase ❑ Other (specify) ________________

☞ **Go to Question 4**

3. If you DO NOT have critical illness insurance, please list reasons for not purchasing (check all that apply)

❑ Don't believe I need it, or didn't want it ❑ Don't have it, but wish I had purchased ❑ Could not afford it
❑ Did not qualify/turned down ❑ Other ___________________________________

4. Over the **PAST 4 weeks**, please describe **how much money was paid** related to **your cancer.** We would like to know about costs paid by **yourself**, **insurance/government** or by **someone else**.

When you paid for something up front which was fully refunded by insurance, list as paid by insurance. Some things may be paid by two or more sources. **Please check all that apply**.

Example: If a medication cost $100 and your insurance paid $80, you would list $20 paid by yourself, and $80 paid by insurance**.**

Have you spent money on prescription drug costs in the PAST 4 weeks (for example, oral chemotherapy, antibiotics, pain medications or anti-nausea medications):

❑ I had no prescription drug costs

	**Amount**	**Don't Know Amount**
☑ Paid by yourself	$ 20	❑
☑ Paid by insurance	$ 80	❑
❑ Paid by other (specify):	$	❑

Other instructions:

Costs paid by the **government**, **industry programs**, **support programs** or **charities** should be recorded in the **'Paid by other'** category.
If you are **unsure** of the exact amount, make your best guess.
If you **don't know** an amount, check the box ("Don't Know Amount").
If you **had no costs** related to each category (for example you were hospitalized for all 4 weeks), check the first box, which states you had no costs.

4a) Have you spent money on prescription drug costs in the PAST 4 weeks (for example, oral chemotherapy, antibiotics, pain medications or anti-nausea medications):

❑ I had no prescription drug costs

	**Amount**	**Don't Know Amount**
❑ Paid by yourself	$	❑
❑ Paid by insurance	$	❑
❑ Paid by other (specify):	$	❑

4b) Have you spent money on in-home healthcare costs in the PAST 4 weeks (for example, nursing, home health aides or physiotherapy):

❑ I had no in-home healthcare costs

	**Amount**	**Don't Know Amount**
❑ Paid by yourself	$	❑
❑ Paid by insurance	$	❑
❑ Paid by other (specify):	$	❑

4c) Have you spent money on homemaking costs in the PAST 4 weeks (for example, cleaning or cooking):

❑ I had no homemaking costs

	**Amount**	**Don't Know Amount**
❑ Paid by yourself	$	❑
❑ Paid by insurance	$	❑
❑ Paid by other (specify):	$	❑

4d) Have you spent money on complementary and alternative therapy costs in the PAST 4 weeks (for example, acupuncture, Chinese medicine, homeopathy, massage, music therapy or naturopathy):

❑ I had no complementary and alternative therapy costs

	Amount	Don't Know Amount
❑ Paid by yourself	$	❑
❑ Paid by insurance	$	❑
❑ Paid by other (specify):	$	❑

4e) Have you spent money on other health professional costs in the PAST 4 weeks (for example, physiotherapy, or counseling):

❑ I had no health professional costs

	Amount	Don't Know Amount
❑ Paid by yourself	$	❑
❑ Paid by insurance	$	❑
❑ Paid by other (specify):	$	❑

4f) Have you spent money on vitamin and supplement costs in the PAST 4 weeks (for example, supplements, special foods or diets):

❑ I had no vitamin and supplement costs

	Amount	Don't Know Amount
❑ Paid by yourself	$	❑
❑ Paid by insurance	$	❑
❑ Paid by other (specify):	$	❑

4g) Have you spent money on family care costs in the PAST 4 weeks (for example, babysitting for children, elder or respite care):

❑ I had no family care costs

	Amount	Don't Know Amount
❑ Paid by yourself	$	❑
❑ Paid by insurance	$	❑
❑ Paid by other (specify):	$	❑

4h) Have you spent money on overnight stays and meal costs in the PAST 4 weeks (for example, hotel or meals in hospital):

❑ I had no accommodation and meal costs

	Amount	Don't Know Amount
❑ Paid by yourself	$	❑
❑ Paid by insurance	$	❑
❑ Paid by other (specify):	$	❑

4i) Have you spent money on devices, supplies, or equipment costs in the PAST 4 weeks (for example, wigs, wheelchair, hearing aids or crutches):

❑ I had no devices, supplies or equipment costs

	Amount	Don't Know Amount
❑ Paid by yourself	$	❑
❑ Paid by insurance	$	❑
❑ Paid by other (specify):	$	❑

4j) Have you spent money on other costs in the PAST 4 weeks (for example, fitness or yoga classes, telephone costs, pagers, and over the counter drugs such as Gravol or Tylenol):	
❑ I had no other costs	
Please list your other costs:	
**Item:**	Dollar Amount $
**Item:**	Dollar Amount $
**Item:**	Dollar Amount $
**Item:**	Dollar Amount $

5. Over the **PAST 4 weeks**, please describe **up to 3 locations** you as a patient have traveled to most frequently or were the longest trips you made for **your HEALTHCARE**, e.g. cancer centre, local hospital, pharmacy

	Example	**Please list up to 3 locations:**		
Locations (List up to 3)	*Hometown Hospital*	**#1:**	**#2:**	**#3:**
**Number of one-way trips in the PAST 4 weeks**	*2*			
**Please tell us the distance in kilometers or miles of a one-way trip.**<br><br>**Or**<br><br>**Please tell your final destination and us from where you started.**	**Distance**: __________ (km or mi)<br><br>OR<br><br>**Start:** *Dundas St. & Main St.*<br><br>**End:** *Bayview Ave & Lawrence Ave.*	**Distance:** __________ (km or mi)<br><br>OR<br><br>**Start:** ____________<br><br>**End:** ____________	**Distance:** __________ (km or mi)<br><br>OR<br><br>**Start:** ____________<br><br>**End:** ____________	**Distance:** __________ (km or mi)<br><br>OR<br><br>**Start:** ____________<br><br>**End:** ____________
**Method of Transport (car, taxi, bus, train, etc.)**	*Car*			
**Parking, Fare or Road Toll (average per trip)**	*$ 25.00*	$	$	$
**Paid for by Insurance or Government**<br><br>**(Please circle)**	Yes – (No)	Yes – No	Yes – No	Yes – No
	20 minutes (without traffic)			

**Average travel time of a one-way trip**				

6. For you and your family, how much **financial difficulty** have the expenses related to **your care** caused in the **PAST 4 weeks?** (For example, *healthcare costs, travel costs and lost income*)

❑ No financial difficulty ❑ Small financial difficulty ❑ Somewhat of a financial difficulty
❑ Large financial difficulty ❑ Worst possible financial difficulty

7. Do you expect to apply for a "medical expenses tax credit" or "financial aid" for your cancer-related costs?
❑ YES tax credit ❑ YES financial aid ❑ NO ❑ I did not know I could apply

8. Do you think you would benefit from financial advice on the following topics (check all that apply)
❑ NO need for financial advice ❑ credit card management ❑ cash flow management
❑ effective savings strategies ❑ eliminating unnecessary expenses ❑ other __________________

9. Did the costs for **your care** require you to do any of the following in the **PAST 4 weeks**?
(Check all that apply)

❑ Use some of your non-registered savings (for example: general savings)
❑ Take a loan (personal loan with family member or friends, bank or mortgage your home)
❑ Use some of your registered savings (for example: RRSP, RESP, RDSP, TFSA)
❑ Move to a less expensive home or cheaper home
❑ Modify/renovate your home to accommodate cancer related issues
❑ Other ways: please specify: ____
❑ Did not have to do any of the above

10. Did the costs related to **your care** result in you deciding to change what you spend your money on in the **PAST 4 weeks**?

❑ **Yes**, the costs **did** affect my spending decisions (Go to question 11)
❑ **No**, the costs **did not** affect my spending decisions (SKIP question 11, Go to question 12)
❑ No costs for my care, in last 4 weeks (SKIP question 11, Go to question 12)

11. Did you **reduce** your spending in the **PAST 4 weeks** because of costs related to your care?
(Below, check all that apply)

❑ **Entertainment, other activities or family events.** For example, postponing vacations, drinking less coffee, fewer dinners at restaurants or stopped sports or music lessons for family members.

❑ **Changes in saving patterns.** For example, smaller contributions to your savings account, education funds, retirement savings or retirement plan.

❑ **Medical services for other family members (parents, spouse or children**). For example, prescription medications, counseling or supplements such as vitamins/minerals.

❑ **Change in your own care.** For example, if you reduced your spending on homecare services for yourself, please check, homecare.
(Check all that apply)

❑ Medications ❑ Family care

- ❑ Homecare
- ❑ Homemaking
- ❑ Complimentary and alternative therapy
- ❑ Vitamins and supplements
- ❑ Accommodation or meals
- ❑ Devices or equipment
- ❑ Other (specify) ____________________________

Part 2: Questions 12 to 14 ask about extra financial costs related to you and your caregi[illegible]

These questions will ask about **you and your caregivers** in the **PAST 4 weeks**. This section includes questions about time away from work. Please list up to 3 caregivers who routinely provide care for you and are working or recently left paid work.

	**Patient**	**Caregiver 1**	**Caregiver 2**	**Caregiver 3**
**Please indicate which caregiver (for example, mother, father, spouse, son or daughter)**				

12. How much time, over the **PAST 4 weeks,** did you and your caregiver(s) take off work to care for you?

In the PAST 4 weeks, **did not work** (was not employed or was retired for the entire 4 weeks) (☑ Check one option per person)				
In the PAST 4 weeks, **stopped work** (quit or leave of absence) (☑ Check one option per person)				
In the PAST 4 weeks, individual **reduced hours of work** (*please indicate average number of hours reduced per week*)	***(# of hours)***	***(# of hours)***	***(# of hours)***	***(# of hours)***
In the PAST 4 weeks, individual **took some time off from work** (*please indicate the number of days off work from 0-28*)	***(# of days)***	***(# of days)***	***(# of days)***	***(# of days)***

13. In the **PAST 4 weeks**, if any person indicated time off from work, was this time away from paid work? (☑ Check all that apply per caregiver)

In PAST 4 weeks, **took no time off work**				
In PAST 4 weeks, **took time off work with full pay** (e.g. vacation time, personal days or sick days)				
In PAST 4 weeks, **took time off work with partial pay**				
In PAST 4 weeks, **took time off work without pay**				

14. Previous to the PAST 4 weeks, have you or a caregiver quit work as a result of your cancer diagnosis? (☑ Check one option per caregiver)

No, did not quit work **previous to the PAST 4 weeks**				

Yes, did quit work **previous to the PAST 4 weeks**				

Part 3: Questions 15 to 30 ask about you, your work, your education and your cancer

15. Year of Birth _________

16. Gender: ❑ Male ❑ Female ❑ Other

17. When was your **FIRST outpatient treatment** (chemotherapy, radiation, surgery) for **your cancer**:

*(If **your cancer** has returned (recurrence), the **FIRST outpatient treatment** date equals the start of treatment for the recurrence)*

____ day (if known) ____ month ____ year **<u>OR</u>** ❑ Don't Know

18. How has your cancer doctor described the goal of treating your cancer?

❑ cure your cancer ❑ slow down the progress of your cancer
❑ relieve symptoms of the cancer ❑ Don't know

19. What is the highest level of schooling you have completed?

❑ No schooling, some elementary school, or completed elementary school
❑ Some high school
❑ Completed high school
❑ Some university or community college
❑ Completed university or community college
❑ Post Graduate (MSc/MBA/PhD) or professional training (MD/LLB/DDS)

20. What was your total family income before taxes in the last year (include wages, salaries and self-employment earnings)?

❑Less than $5,000
❑$5,000- $9,999
❑$10,000- $14,999
❑$15,000- $19,999
❑$20,000-$29,999
❑$30,000-$39,999
❑$40,000-$49,000
❑$50,000- $59,999
❑$60,000-$79,999
❑$80,000-$99,000
❑More than $100,000
❑Don't Know

21. What percentage of family income was earned by you (the patient) last year?

❑ None ❑ 1-24% ❑ 25-49% ❑ 50-74% ❑ 75-99% ❑ 100%

22. Have you been the primary caregiver (significant disruption from usual daily routine) for a cancer patient in the past?

❑ No ❑ Yes, in the last year ❑ Yes, in the last 5 years ❑ Yes, but more than 5 years ago

23. Marital Status:

❑ Married ❑ Common Law ❑ Single (never married)

❑ Widowed ❑ Separated ❑ Divorced

24. How many other people do you share your home with (do not include people who are only visiting):

❑ Live alone (∗ **Go to Question 26**) ❑ Myself and one other

❑ 2 others ❑ 3 others ❑ More than 3 others

25. Are these people you share your home with:

❑ Family ❑ Friends ❑ Both Family and Friends

26. Postal code OR City or Town where you live ______________________________

27. Over the past 4 weeks did you receive any of the following (check all that apply):

Chemotherapy	❑ NO	❑ YES…	If yes	❑ 1 of 4 wks	❑ 2 of 4 wks	❑ 3 of 4 wks	❑ Every wk
Hormone therapy	❑ NO	❑ YES…	If yes	❑ 1 of 4 wks	❑ 2 of 4 wks	❑ 3 of 4 wks	❑ Every wk
Radiation	❑ NO	❑ YES…	If yes	❑ 1 of 4 wks	❑ 2 of 4 wks	❑ 3 of 4 wks	❑ Every wk
Active surveillance	❑ NO	❑ YES					
Surgery	❑ NO	❑ YES					

28. In terms of your current cancer, how would you describe today's treatment

❑ Near the beginning of your treatment schedule
❑ Near the middle of your treatment schedule
❑ Near the end of your treatment schedule
❑ Don't know

29. How would you rate your current health?

❑ Excellent ❑ Very good ❑ Good ❑ Fair ❑ Poor

30. What do you do for a living:

❑ *Full time work: Specify* _______________ ❑*Part time work: Specify* _____________

❑ *Retired* ❑ *Homemaker* ❑ *Unemployed* ❑ *Student*

Part 4: Question 31 asks about the cost of cancer not discussed in this survey

31. Additional comments about other expenses or decisions related to your cancer (example might include: educational, social, spending more money on nutrition, anticipated longer term costs such as major home renovations, a caregiver that started working or got a second job, or the impact on your career).

____

____

____

____

Thank you for taking the time to complete this questionnaire.
